# Supplementary material for: RBD-specific antibody response after two doses of different SARS-CoV-2 vaccines during the mass vaccination campaign in Mongolia
Source: PLoS One. 2023 Dec 8;18(12):e0295167. doi: 10.1371/journal.pone.0295167 (PMC10707641; doi:10.1371/journal.pone.0295167)
Supplement: S1 Table — (DOCX) [file pone.0295167.s001.docx]

**Supplementary Table 1. The sociodemographic pattern of frontline employees from different rural sites**

| **Characteristics of participants** | **Bulgan**  **(n = 122)** | **Darkhan-Uul**  **(n = 50)** | **Dornod**  **(n = 97)** | **Orkhon**  **(n = 42)** |
| --- | --- | --- | --- | --- |
| **Sex, count (percent)** |  |  |  |  |
| **Males** | 21 (17.2) | 6 (12.0) | 11 (11.3) | 9 (21.4) |
| **Females** | 101 (82.8) | 44 (88.0) | 86 (88.7) | 33 (78.6) |
| **Age (years)** |  |  |  |  |
| **Mean (M ± SD)** | 38.7 ± 12.0 | 40.5 ± 14.0 | 40.7 ± 10.0 | 38.1 ± 9.8 |
| **CI 95** | 36.6 – 40.9 | 36.5 – 44.5 | 38.7 - 42.7 | 35.1 - 40.8 |
| **Min. – Max.** | 19 - 59 | 18 - 56 | 23 - 57 | 23 - 60 |

Abbreviations: M, mean; SD, standard deviation; CI95, confidence interval of 95%; Min. – Max., lowest and highest values
