# Supplementary material for: RBD-specific antibody response after two doses of different SARS-CoV-2 vaccines during the mass vaccination campaign in Mongolia
Source: PLoS One. 2023 Dec 8;18(12):e0295167. doi: 10.1371/journal.pone.0295167 (PMC10707641; doi:10.1371/journal.pone.0295167)
Supplement: Attachment — Submitted filename: Response to reviwers.docx [file pone.0295167.s002.docx]

Dear Editor,

We are very grateful to you for allowing us to revise and submit the manuscript.

Firstly, I would like to inform you that we analyzed all data thoroughly after we submitted the manuscript in early February 2023. And we found 311 participants who were excluded from the analysis due to “not complete” data in primary research units. Many participants were excluded due to insufficient personal information (e.g., unclear ID number, home address, and change in health condition after the first dose). Once we had enough time and no covid restrictions, we connected with participants and clarified their missing personal information. Finally, we added data from 254 participants for the analysis. As a result, some values of our study have to be changed. But these changes were not principal, and the main conclusions remained unchanged. Also, we added four more authors to the authorship list.

You recommended depositing our laboratory protocols in protocols.io. We used the Anti-SARS-CoV-2 S-RBD protein Human IgG ELISA Kit (Cat no: KE30003) and Anti-SARS-CoV-2 S-RBD protein Human IgM ELISA Kit (Cat no: KE30004) from Proteintech, USA. These are research use-only kits, and the Material Safety Data Sheet of these products is available on the manufacturer’s website. We received instructions for the use of these products with kits from the manufacturer and I think it is only for buyers.

The manuscript was revised and corrected according to the journal’s requirements and reviewers’ comments. These include:

Journal’s requirement:

1. Please ensure that your manuscript meets PLOS ONE's style requirements, including those for file naming.

Answer:

The revision was done according to PLOS ONE’s style requirements.

2. Did participants provide their written or verbal informed consent to participate in this study?

Answer:

The current ethics statement was provided with the written informed consent of participants.

3. We suggest you thoroughly copyedit your manuscript for language usage, spelling, and grammar.

Answer:

The manuscript was edited by an experienced English speaker. Dr. Ryenchindorj Erkhembayar from the Graduate School of our university provided an English edition of the paper.

4. We note that you have indicated that data from this study are available upon request.
a) If there are ethical or legal restrictions on sharing a de-identified data set, please explain them in detail.

Answer:

We can share the data of participants after withdrawing their name and ID number (so-called Number of Register for each citizen of Mongolia). However, we enrolled 68 subjects with confirmed HIV infection. According to the Law of Mongolia named “About Prevention of Human Immunodeficiency Virus Infection and Acquired Immunodeficiency Syndrome” issued on 13 December 2012 by the Parliament of Mongolia, we are not able to share their data. You can find the Law in the Mongolian language by the link <https://legalinfo.mn/mn/detail?lawId=8913>

5. Your ethics statement should only appear in the Methods section of your manuscript.

The Ethics Statement was placed in the Methods section

Answer to Review Comments

Reviewer #1: I find all the parts of the paper correctly written from background to the conclusion and of high quality. Methods are clearly explained. Sample size is proper for this kind of research and enables conclusions to the wider population. Results are clear and correct. Statistical analyses is proper for this kind of research. Discussion is sound and comments various research with comparison to findings of this paper. Limitations are clearly stated. All the data needed to understand the paper are presented in clear and common fashion. English is standard and paper is presented well. I can only extend my congratulations to the authors and my gratitude to the editor for the possibility to review this paper. I have no suggestions to improve this paper.

Answer: Thank you

Reviewer #2: Thank you for inviting me to review this very interesting manuscript, which provides insight into the vaccination roll out in Mongolia. This study shows more granular clinical information than prior studies in this country and focuses specifically on those who are 'naive' to prior infection. Given these two novel features there are some major considerations:

1. Which type of vaccine an individual received was based on which group they were in. High-risk healthcare workers received BBIBP-CorV. Priority groups received vaccination ‘following the arrival of further doses’, presumably BBIBP? (Note in discussion it says these some of this group received Pfizer - Line 189). Pfizer, ChAdOx1, Sputnik became available later when vaccination rollout was expanded to the whole population. Therefore comparison of types of vaccine has major confounders including age (high risk included those over 65 years old) and co-morbidities. These confounders are well described as impacting antibody response, yet the analysis does not account for this when comparing antibody response by vaccine type. It is significantly understated in the authors discussion, which is surprising.

Answer:

Date of arrivals for four types of vaccines:

AstraZeneca - February 23, 2021, and March 12, 2023.

<https://www.who.int/mongolia/news/detail/23-02-2021-covid-19-vaccination-rollout-in-mongolia>

<https://www.unicef.org/mongolia/press-releases/mongolia-welcomes-first-batch-covid-19-vaccines-covax-facility>

Sinopharm - February 23, 2021, and April 20, 2021

<https://thediplomat.com/2021/05/how-mongolia-made-the-most-of-vaccine-diplomacy>

<https://asia.nikkei.com/Spotlight/Coronavirus/COVID-vaccines/Mongolia-resumes-vaccinations-as-worst-outbreak-rolls-on>

Sputnik V - February 27, 2021, and April 30, 2021

<https://news.mn/en/795436/>

<https://www.capitalsinitiative.org/2021/06/15/covid-19-vaccine-rollout-accelerated-to-all-above-18-years-eligible-in-ulaanbaatar/>

Pfizer - June 17, 2021

<https://www.unicef.org/mongolia/press-releases/over-84000-pfizer-vaccines-arrive-mongolia-first-batch-25-million-doses#:~:text=Press%20release-,Over%2084%2C000%20Pfizer%20vaccines%20arrive%20in%20Mongolia%2C%20the,batch%20of%202.5%20million%20doses.&text=June%2016%2C%202021%20Ulaanbaatar%20%2D%20Over,batch%20of%202.5%20million%20doses>.

As you see, AstraZeneca and Sinopharm vaccines arrived before started mass vaccination, on February 23, 2021. Frontline workers started the vaccination with AstraZeneca, but there were only 14.4 thousand doses of this vaccine. The arrival of the next batch of AstraZeneca was delayed. The Ministry of Health decided to continue the vaccination of frontline employees with the Sinopharm vaccine. So, we have participants who received both vaccines in this group. It is my fault; I did not check the citation source accurately. I corrected the text as follows: “Mongolia received the first batch of the inactivated BBIBP-CorV vaccine from Sinopharm, China, and the non-replicating viral vector vaccines Oxford-AstraZeneca, from India and began immunization in high-risk healthcare workers”

Choice of vaccine type

Citizens of Mongolia had the opportunity to choose the type of vaccine that was available in the immunization unit.

Age and comorbidity impact

All immunocompromised patients or patients who passed immunosuppressive therapy (SAD and Cancer patients) and PLWHA were immunized only with the Pfizer vaccine. 32 out of 48 elderlies received the Sinopharm vaccine.

Additionally, I have performed ROC analysis of age by seroconversion in vaccine-type groups and found a significant impact only in the Sinopharm group. This finding was added to Figure 3 and explained in the text.

2. The study cohort have been selected on having no prior infection to the first dose. It is unclear how the authors accounted for infection between vaccinations, which again will influence antibody response. If the authors have N-antibody available, they should include this in their analysis. If they don't, this should again be discussed in detail in their discussion and consider how this may impact their findings (e.g., probability of infection in that time window based on transmission risk at the time - note some of this study was conducted during a lockdown).

Answer:

I agree with you. We did not provide serological or PCR evidence for infection between shots. The serological investigation was not rational because the Sinopharm vaccine is able to stimulate the production of N-antibodies. We used the following two database to exclude cases of natural infection: 1) we collected information regarding possible natural infections which may occur during the observation by filling out a questionnaire when vaccinees were invited for data and sample collection in days 21 – 28 after second dose. 2) In Mongolia, all new PCR confirmed cases of SARS-CoV-2 infection were registered in the “Gerege” system <https://gerege.mn/en/home>. This system was connected directly to the database of the Ministry of Health. We have checked all suspicious cases in this database later for exclusion of natural infection from the analysis.

I have added a correction in Figure 1 regarding your comment.

3. It would be useful to know how exactly participants in the third group were selected (e.g., household based, clinic based...etc). How they were selected, and how it differs to the other groups, may highlight selection bias and would need to be explored in the discussion. I presume selection of group 2 was base done on hospital records? Again, this is not clear.

Answer:

Participants in the third group were randomly selected in family doctor’s centers. Vaccine administration units were organized in family doctor centers. Family doctor’s units were randomly selected in two of six urban districts of Ulaanbaatar city. Ulaanbaatar is divided into nine [düüregs](https://en.wikipedia.org/wiki/D%C3%BC%C3%BCreg) (usually translated as *districts,* six districts located in the urban areas, but three districts located in suburban areas), which are further subdivided into [khoroos](https://en.wikipedia.org/wiki/Khoroo) (most often translated as *subdistricts*). link: <https://en.wikipedia.org/wiki/Administrative_divisions_of_Mongolia#:~:text=Ulaanbaatar%20is%20divided%20into%20nine,%2C%20microdistrict%20or%20simply%20district>). An explanation according to a selection of participants from group two and three was done in the Methods section.

More specific queries include:

Line 48: Unclear what ‘locally grown cases’ means? Cases that had no clear link to migration?

Answer:

This phrase was cited directly from the reference. I corrected it as “local cases increased gradually”.

Table 1: What are ‘red-line’ and ‘yellow-line’ facilities? Can you please provide explanation in caption?

Answer:

I replaced the word “line” with the word “label”. An explanation of the employees of “red label” and “yellow label” facilities was updated in the caption of the Table 1.

Table 2: Please include use of ANOVA in the statistical analysis part of methods.

Answer:

The use of ANOVA was included in the Statistical Analysis subsection of the Methods section.

Line 210: Can you clarify definition of ‘sociodemographic’ pattern please? It would be interesting to know age distribution, proportion of sex and proportion of co-morbidities between the rural areas and rural vs urban.

Answer:

Here, the term “sociodemographic pattern” was used for frontline employees of different rural sites. This data was shown in newly added S1 Table regarding your comment.

Sincerely yours

Tsogtsaikan Sandag

MD, PhD, Professor

Department of Immunology, School of Biomedicine,

Mongolian National University of Medical Sciences,

Zorig street 3 Sukhbaatar district Ulaanbaatar 14210

Mongolia

email: tsogtsaikhan.s@mnums.edu.mn
